# A Social Media Intervention to Increase Caregiver Knowledge of Early Infant Communication: Multiple Methods Intervention Development and Pilot Study

**DOI:** 10.2196/93171

**Published:** 2026-09-30

**Authors:** Mollie Romano, Aliya Junaideen, Frances Baehman, Katherine Dale, Arienne Ferchaud, Diana Lucia Abarca Sasser, Ashley Sellers

**Affiliations:** 1 School of Communication Science and Disorders Florida State University Tallahassee, FL United States; 2 School of Communication Florida State University Tallahassee, FL United States; 3 School of Communication Science and Disorders University of Central Florida Orlando, FL United States

**Keywords:** parenting, infant, social media, language development, mHealth, early intervention

## Abstract

**Background:**

Greater caregiver knowledge of early language development may support more reciprocal interactions with infants and improved child language outcomes. Short-form video interventions on social media platforms like Instagram and TikTok could be a scalable, cost-effective way to increase caregiver knowledge.

**Objective:**

The objective of this investigation is to describe the development and testing of a version of the BabyTok Project, a social media–based intervention for caregivers of very young infants under 3 months of age.

**Methods:**

We used a multiple-method, pre-post quasi-experimental design to examine the feasibility, acceptability, and initial evidence of effectiveness of the BabyTok Project through a knowledge measure called the SPEAK-R (Survey of Parent/Provider Expectations and Knowledge–Research).

**Results:**

Engagement metrics, participants’ postintervention surveys, and feedback support the feasibility and acceptability of the intervention. Statistically significant scores on the SPEAK-R offer initial indications of change in caregiver knowledge.

**Conclusions:**

The BabyTok Project has multiple demonstrations of its feasibility and acceptability and shows promise as a means to increase caregiver knowledge of early infant communication development and strategies to enhance children’s learning. Interventions like the BabyTok Project could be a scalable way to promote optimal child language outcomes.

## Introduction

### Early Language Development

Infants are born interactional partners, and their earliest experiences with caregivers set their trajectory for language acquisition in the years that follow [1]. Infants who experience frequent, responsive, back-and-forth interactions go on to have a better understanding of language, larger spoken vocabularies, more complex grammar, and stronger reading, writing, social-emotional, and economic outcomes later in life [2-5]. The impact of these reciprocal exchanges is also visible in children’s neuroanatomy [1,4,5]. Infants who experience more back-and-forth vocal turns with caregivers in the first year of life have denser white matter tracts in areas of the brain that are responsible for understanding and production of language, suggesting that the cumulative impact of early interactions begins in the first year of life [1]. For infants whose families experience economic hardship, responsive interactions serve as a protective factor against the wide-ranging negative neurodevelopmental consequences of poverty on early learning [6,7].

Promoting positive, responsive early interactions is a well-established way to improve children’s language learning in communities that are experiencing poverty [8,9]. However, most interventions that aim to support caregivers to engage with their children are limited in two ways. First, many interventions begin later in toddlerhood rather than the earliest months of life [8]. Although these programs are still meaningful during toddlerhood, infants’ brains develop networks that specialize in language from the very first days of life [1]. As such, promoting optimal child language learning should begin at or even prior to birth. Second, most interventions that aim to enhance the frequency and quality of infant-caregiver interactions use a coaching model in which a professional observes, guides, and gives feedback to caregivers. These interventions have a long history of evidence [10-12]. However, scaling preventative coaching models population-wide is difficult due to the cost of implementation [8], instability in funding, and variability in what home visiting programs operate in each state.

### Light-Touch Interventions

Because coaching models are costly, researchers suggest the need for scalable, population-level interventions that increase caregiver knowledge of early language learning [8]. Compared to other areas of health sciences like smoking cessation [13], HIV prevention [14], or breastfeeding promotion [15], there have been relatively few investigations of mobile health efforts to promote optimal child language development. Research in this area is critical as approximately 30% of infants and toddlers whose families have low incomes are late talkers who may need ongoing and more intensive language interventions when they reach school [16]. One way to increase child language outcomes is to increase what caregivers know about early language learning [17]. Caregiver knowledge of early development is linked to more frequent and more responsive language interactions, so building caregiver knowledge is an important avenue for universal interventions at the population level [17]. Below, we highlight 2 mobile health, light-touch interventions to enhance caregiver knowledge and strategy use to promote their child’s early language learning.

Háblame Bebé is a free Spanish-English mobile app designed to promote early bilingual language development among infants and toddlers by supporting parents to provide rich, engaging verbal input [18]. Early evaluations of the app indicate promising outcomes for enhancing parents’ knowledge and language-supportive behaviors [18-20]. In a quasi-experimental study, researchers found that mothers who used the app increased the amount and quality of their language directed toward their children during play and shared reading [18]. Postintervention, participant mothers produced more total words, asked more questions, and demonstrated a stronger sense of pride and confidence in using Spanish with their children [18]. A subsequent randomized controlled trial assessed the app’s impacts on both caregiver behaviors and early child language outcomes when implemented in real-world conditions without researchers’ support to access and use the app [20]. Although the trial did not find statistically significant group differences in standardized child language scores, effect sizes favored the intervention group. Caregivers also reported high usability, acceptability, and satisfaction levels with the app as a light-touch intervention, highlighting its accessibility and cultural relevance [21].

Caregivers of infants in the United States widely use the internet social media platforms for information seeking about their child’s health and development [22-25]. Unlike Háblame Bebé, which uses a standalone app, we created an intervention called the BabyTok Project to connect caregivers (eg, parents and infant/toddler teachers) [26,27] with evidence-based information via short-form videos on TikTok. Through initial testing, both sets of caregivers endorsed the intervention as feasible and acceptable, and caregivers reported that they learned new information that helped shape interactions with infants and toddlers in their care. These studies also provide information about engagement metrics for this type of intervention. While the 2025 investigation indicated positive directional change in caregiver knowledge, the gains were not statistically significant, perhaps due to the underpowered nature of the feasibility study and the short-term nature of the intervention. In the present iteration of the BabyTok Project, we extend the intervention to caregivers whose infants are under 3 months old to maximize the impact of responsive interactions with a larger sample size, and we lengthened the intervention to approximately 4 months.

### Purpose of the Study

The purpose of this study was to describe the development of a social media–based intervention specific to the needs of caregivers of young infants (under 3 months) and to conduct a pre-post pilot test of the BabyTok Project to explore whether there are gains in caregivers’ knowledge of early language development. We also aim to explore caregivers’ perceptions of the intervention content and delivery to directly inform a larger-scale randomized controlled trial.

## Methods

### Study Design

This study used multiple methods, qualitative methods through focus groups during the development phase and a pre-post quasi-experimental design during the pilot, to determine feasibility and acceptance of the BabyTok Project, a 22-week intervention using social media to promote child language development. Data were collected to help evaluate user activity and engagement within the app, short-term effects on caregivers, and overall usability. Data were also used to support future adaptations and improvements to the intervention.

### Intervention: The BabyTok Project

#### Content Development

Prior to the intervention, we created sample videos to share with focus groups to gain caregiver input before proceeding with full video development of the intervention. The fourth and fifth authors helped to design focus group protocols to learn more about how caregivers use social media, what types of content they search for related to their infants, and what makes a video catch and keep their attention. We also showed samples of the BabyTok Project videos to solicit feedback on style and content. Additionally, at the end of each focus group, we provided a list of video topic ideas and invited participants to share their opinions to help us understand more about what topics related to language and communication are most important to caregivers of infants. The focus group participants were recruited via an ad on Instagram, which ran for 4 weeks from mid-December 2023 to mid-January 2024. The ad used a URL code that sent potential participants to a Qualtrics form, which requested name and contact information. Those who gave their information were contacted via email and were asked the age of their infant and their caregiver status. Then, they were emailed a link to a Qualtrics survey, which included demographics and income-related questions. To be placed in a focus group, participants needed to be a caregiver of a child aged 0 to 6 months and qualify for social services such as Medicaid, housing assistance, TANF (Temporary Assistance to Needy Families), WIC (Women, Infants and Children), and/or food pantry/other goods. We conducted two focus groups with a total of 8 participants. The first focus group consisted of 3 participants who all reported being parents of infants, male, between the ages of 29 and 32 years, and Black or African American; 2 reported having a bachelor’s degree and 1 an associate’s degree; 2 reported living in an urban area and 1 reported living in a rural area. The reported income distribution among them was between US $40,000 and US $79,999. All 3 participants reported speaking English. The second focus group consisted of 5 participants who all reported being parents of infants, female, and between the ages of 24 and 32 years. Four participants reported being Black and 1 reported being Black and Hispanic/Latino; 1 reported having a bachelor’s degree, 2 reported some college/associate degree, and 2 reported having a high school diploma; 3 reported living in a suburban area, 1 reported living in a rural area, and 1 reported living in a small town. The reported income distribution among them was wide, with reported earnings of US $20,000-29,999, US $30,000-39,999, US $70,000-79,999, and US $80,000+.

The groups were conducted via Zoom by the sixth author and were video- and audio-recorded. The third author and a trained undergraduate assistant attended the groups to help with any technical difficulties and to take notes. Participant responses were recorded via note-taking and read back to them at the end of each session to verify their answers. At the conclusion of the groups, one researcher synthesized the notes and asked for feedback from the other researchers on the team. The synthesized notes were also emailed to the participants for feedback to ensure that we described their opinions accurately. Opinions gathered from the focus groups regarding content they tend to watch and follow on social media indicated that the trustworthiness of content and content creators is of high importance. In addition, the focus groups discussed the importance of content being relatable and enjoyable to watch, with content that grabs your attention quickly described as preferable. The groups agreed that they scroll past content that does not have an immediate hook. When shown the sample videos, they overwhelmingly preferred videos with real caregivers and their babies. Participants also preferred videos with the speaker talking directly to the camera, videos with movement and some music but not too loud to be distracting, and videos with captions. The groups agreed that they enjoyed the educational content of the videos and that the information provided was valuable. Some critiques of the videos were that some were too fast and hard to follow, and the volume of background music was too loud. One focus group participant shared that some of the videos felt overproduced and more like an advertisement from a nongovernmental organization, and other focus group participants agreed. When asked for their opinions on topics they would like to see in future videos, participants were interested in information on children with delays and sign language for babies. This feedback from the focus groups was considered and integrated into the creation of the social media content for the intervention phase of the research. Participants were each emailed a US $50 virtual gift card at the conclusion of the focus groups.

#### Content Description

The project’s fully developed content included 48 videos, each about 1 minute long, all posted on Instagram Reels. All videos were filmed and edited by a professional videographer, with the first author serving as the narrator. Each video featured the first author as well as B-roll clips of several families who volunteered to model for videos. The project also included 13 still posts with facts, affirmations, or questions as prompts to create a more natural, community feel, encouraging parents to engage, comment, and share as they would on a typical Instagram page. Three videos were also reposted as examples to illustrate concepts and activities discussed. Video content was created using the best available research on early language development and caregiver interactions [26,27]. The first video served as an introduction, guiding parents on what to expect during the study and introducing the first author’s background as a speech-language pathologist, professor, researcher, and mother. The remaining videos covered the following topics: brain development, affirmations, early cues, routines, books, and specific strategies. For more detailed information on video content, see Table 1.

**Table 1:** BabyTok Project content descriptions (n=64).

Topic	Posts/videos, n (%)	Purpose
Brain development	5 (8)	These videos teach caregivers about early brain development and how infants’ brains are primed for learning language from birth.
Affirmations	5 (8)	Affirmation videos were shared to provide encouragement, confidence, and emotional support to caregivers.
Early cues	6 (9)	Babies communicate as soon as they are born through cries, fusses, and their movements. These videos help parents to recognize and interpret their baby’s cues, give tips on how to respond, and boost their future communication skills.
Routines	4 (6)	Everyday routines help establish a sense of predictability that supports babies’ learning. These videos help caregivers build routines by providing guidance about how to build in predictability around caregiving tasks, book sharing, and more.
Books	6 (9)	These videos highlight the benefits of reading even to young infants, and we provide tips on effective strategies like following children’s attention/gaze and selecting engaging books for infants.
Specific strategies	14 (22)	These videos share ways caregivers can support language development through everyday interactions. They cover strategies like infant-directed speech (parentese), modeling language, using baby signs, and playtime tips to encourage tracking and reaching.
Myth busting	8 (13)	This section of videos addresses common concerns, developmental milestones, and misconceptions during a baby’s first year, covering key topics like bilingualism and screen time.
Still posts	13 (20)	Still posts were used to provide announcements to participants, informative facts, affirmations, and question prompts to encourage participant engagement.
Reposts	3 (5)	TikTok reposts served as examples of previously discussed concepts (eg, signs to use or songs to sing) as well as sharing helpful tips from other parents. Reposts were also used to capture a greater demographic range of caregivers featured in the videos.

### Fraud Prevention

Given the virtual nature of this study, we implemented a fraud prevention strategy to ensure data integrity and prevent masqueraders (individuals or bots who submit fraudulent responses) from participating in the study. The survey was completed on Qualtrics and received 308 responses. The first line of defense to prevent fraudulent responses was adding the Qualtrics Re-captcha feature to the initial 2-question prescreener. This feature did not detect all potential fraud. Therefore, to ensure that our participants were real individuals and to prevent ineligible participants from entering the study, we used a fraudulent response detection process, which included filtering out Qualtrics’s identified bots and incomplete surveys, removing respondents with duplicate IP addresses and locations outside of the United States, and removing respondents who gave unintelligible or inappropriate answers to open-ended questions [28]. To provide extra verification, emails were sent with the following questions: (1) Are you a regular caregiver of a child? (2) If so, please provide the age of your child in months. If responses were inconsistent with survey data, these individuals were deemed ineligible to participate in the study.

### Recruitment

Recruitment for this study began in the summer of 2024 through flyers that were distributed both in person and via email to local pediatric and obstetrics/gynecology clinics across the state of Florida. The flyer was also shared on social media platforms, including Instagram, TikTok, and Facebook. It featured a link and QR code that directed individuals to a survey containing 2 prescreening questions to verify caregiver eligibility. To be eligible, participants needed to either be pregnant and due on or before August 15, 2024, or have a child up to 3 months old, and qualify for social services such as Medicaid, housing assistance, TANF, WIC, and/or food pantry/other goods. Participants who were determined eligible were routed to a consent form to provide consent to the institutional review board–approved study. The recruitment period lasted 2 months.

### Procedures

Once participants provided consent, they were sent an email requesting verification of their email address, cell phone number, Instagram handle, and their child’s date of birth. After this information was verified for consistency with the demographic information, participants were sent a follow-up email that included links to the pretest measures, along with instructions for completing them. At this point, they were also asked to follow the study’s Instagram page. Pretest measures included the SPEAK-R (Survey of Parent/Provider Expectations and Knowledge–Research) and demographic forms, which participants completed online via the links provided.

Once the pretest measures were completed and it was confirmed that all participants were following the social media page, researchers began sharing videos and still posts approximately 3 times per week. Participants were asked to watch and like posts to track content engagement. While commenting was encouraged, it was not prompted. Participants received reminders every other week via direct message through the Instagram app, email, or text messages from the research team to watch videos and like them. Following the intervention, participants completed the posttest measures, which included the SPEAK-R and a postintervention survey, all of which were completed online.

### Ethical Considerations

All participants provided implied consent for the institutional review board–approved study (Florida State University Study #00005223) through an online interest form with a waiver of documentation of consent. Participants received up to US $200 in gift cards as compensation. Compensation was distributed via email after pretest data collection (US $100) and the social media intervention and posttest data collection (US $100). All participants were assigned an alphanumeric code for all data files. One single Microsoft Excel file linked personal information to the code. The linking file and deidentified files are kept in an encrypted Microsoft Teams folder hosted by the university.

### Measures

#### SPEAK-R

Similar to the prior BabyTok Project studies with infant/toddler teachers and caregivers of toddlers [26,27], the SPEAK-R was used as a tool to quantify changes in parents’ perceptions of their understanding of early childhood language development. The SPEAK-R is a 22-item, self-administered questionnaire designed to assess knowledge of and beliefs about early childhood cognitive and language development [29]. The questionnaire consists of 4 multiple-choice items, 14 Likert scale items scored as *definitely true* (0) to *definitely not true* (4), and 4 Likert scale items reverse-scored as *definitely not true* (0) to *definitely true* (4). The questions focus on six topics: (1) bilingualism, (2) early exposure, (3) media use for child learning, (4) nature vs nurture, (5) sensitivity and responsiveness, and (6) talking and reading. The SPEAK survey was developed and tested on multiple population samples that included caregivers with low incomes [29]. During development, the SPEAK showed significant, positive correlations between scores and level of education, receptive language skills, and quality of language in the home environment [29]. During field testing, the SPEAK-R was proven reliable with a Cronbach α score of 0.92 [29]. The tool also has a 0.24 correlation (*P*<.001) with the 10-item version of the Knowledge of Infant Development Inventory (KIDI) as evidence of its concurrent validity [30]. We administered the SPEAK-R via an online survey at entry and after the 22-week intervention. The measure was gathered and scored using REDCap, and the data were imported into SPSS for analysis.

#### Instagram Engagement Metrics

For the purposes of this study, we define engagement as “specific interaction and usage patterns” [31] with the app. Prior to the study, we opted to use a private account to help maintain a level of confidentiality for participants. Private Instagram account owners do not have access to data metrics, called Instagram Insights, which track views, impressions, saves, shares, and watch-through rate [32]. To obtain data regarding the participants’ use of the BabyTok Project intervention, participants were asked to “like” posts to indicate viewing. As such, these metrics do not reflect an “organic” pattern of liking, but rather, as an indication that they interacted with the content. Participants were not specifically asked to comment; however, we gathered data on comments per post and per participant as a measure of engagement with the intervention. Data were collected by reviewing each post and documenting the number of likes and comments per post and per participant. After the study was completed, we analyzed the total sum of likes and comments per post and the total sum of likes and comments per participant across the 22 weeks of the intervention and calculated the mean, SD, and range for each. Additionally, feedback regarding the intervention was analyzed by reviewing and coding individual comments for each post.

#### Caregiver Postsurvey

To analyze the acceptability and feasibility of the intervention, we administered an online survey using the Qualtrics platform after the completion of the intervention phase. The postsurvey consisted of 13 Likert scale questions with 5 possible answer choices ranging from *strongly agree* to *strongly disagree*, 1 multiple-choice question about the most helpful content, and 3 short-answer questions regarding takeaways from the content, feedback regarding the content or delivery, and input on future content. Answers to the Likert scale and multiple-choice questions were gathered and analyzed for patterns. Short-answer questions were gathered and analyzed for patterns and themes.

#### Quantitative Data Analysis

##### SPEAK-R

We used descriptive statistics (mean and SD) and a paired-samples *t* test in R to describe changes in caregivers’ knowledge and beliefs about early childhood cognitive and language development as measured by the SPEAK-R. Cohen *d* was calculated for the paired-samples comparison by dividing the mean difference score by the standard deviation of the difference scores [33].

##### Instagram

Instagram data were collected using a simple count of likes and comments per video and per participant. We used a descriptive quantitative analysis to show the means and ranges of Instagram engagement metrics.

#### Qualitative Data Analysis

##### Instagram

Instagram comments were analyzed using the thematic analysis method prescribed by Brooks et al [34]. The second and third authors explored the data separately, and through an iterative process of meetings, constant comparisons, and continued data exploration, common and interesting themes emerged. Through consensus, the themes were labeled as codes, and at the conclusion of this analysis, a simple count of each code was collected to understand more about the engagement of the participants. Throughout this analysis process, the primary author was consulted to ensure the quality of data coding.

As with the previous BabyTok study [27], the data analysis team took several steps to ensure the trustworthiness of the data. To increase the trustworthiness of our findings, our approach included immersion with the data, independent coding with discrepancies resolved through consensus, debriefing with the primary author, and cross-checking for coding reliability [34]. To increase transferability, we provided definitions and several examples of participant comments. To ensure dependability of the data, we kept detailed records and an audit trail.

##### Caregiver Postsurvey

The responses to the short-answer questions in the postsurvey were also analyzed using the template analysis method described in Brooks et al [34]. The second and third authors followed the same process for analyzing the data that was followed for analyzing the Instagram comments. Throughout this process, the first author was consulted to ensure the quality of data coding. To ensure the trustworthiness of the data, we followed the same procedures outlined by Nowell et al [35] as described above in the qualitative analysis of the Instagram comments.

## Results

### Participants

After the verification and fraud prevention processes were completed, a total of 31 participants across the United States were included in the study. Among the participants, there were 27 mothers and 4 fathers. There were 15 participants in the 20-29 years age range, 15 in the 30-39 years age range, and 1 that was 40-49 years old. Of the total participants, 10 were White, 16 were Black, 2 were Hispanic/Latino, 2 were Hispanic/Latino-White, and 1 was Black, White, and Hispanic/Latino. All 31 participants spoke English, 4 spoke both English and Spanish, and 1 spoke both English and Yoruba. For more demographic information, see Table 2.

**Table 2:** Participant demographic information (n=31).

Characteristics			Participants, n (%)
**Relation to child**			
	Mother	27 (87)	
	Father	4 (13)	
**Age in years**			
	20-29	15 (48)	
	30-39	15 (48)	
	40-49	1 (3)	
**Race/ethnicity**			
	White	10 (32)	
	Black	16 (52)	
	Hispanic/Latino	2 (6)	
	Hispanic/Latino-White	2 (6)	
	Black, White, and Hispanic/Latino	1 (3)	
**Languages spoken**			
	English	31 (100)	
	English/Spanish	4 (13)	
	English/Yoruba	1 (3)	
**Educational level**			
	High school diploma or GED^a^	3 (10)	
	Some college	3 (10)	
	Associate degree (AA; AS)	5 (16)	
	Bachelor’s degree (BA; BS)	16 (52)	
	Master’s degree	3 (10)	
	Doctorate degree (PhD)	1 (3)	
**Estimated household income (in US$)**			
	Less than $10,000	1 (3)	
	$10,000-19,999	0 (0)	
	$20,000-29,999	3 (10)	
	$30,000-39,999	3 (10)	
	$40,000-49,999	5 (15)	
	$50,000-59,999	7 (23)	
	$60,000-69,999	4 (13)	
	$70,000-79,999	4 (13)	
	$80,000 or more	4 (13)	

^a^Graduation Education Development.

### Quantitative Results

#### SPEAK-R

Postintervention scores (mean 51.26, SD 12.52) were significantly higher than preintervention scores (mean 44.58, SD 11.37), t_30_=4.54, *P*<.001, 95% CI 3.68-9.68, indicating an improvement in SPEAK-R knowledge scores. The effect size was large (*d*=0.82; Table 3).

**Table 3:** Paired-samples t test for pre- and posttest scores (n=31)^a^.

SPEAK-R	Mean (SD)
Pretest	44.58 (11.37)
Posttest	51.26 (12.52)
Difference	6.68 (8.18)

^a^t_30_=4.54; *P*<.001; 95% CI 3.68-9.68; Cohen *d*=0.82.

#### Instagram Engagement

To ascertain which posts (videos and/or still posts) generated the most engagement, we examined the number of likes per post and the number of comments per post. Engagement rates were slightly higher for video content compared to still posts. On average, videos received 27 likes and 6 comments per post, compared to 24 likes and 5 comments per post for still posts. A video about postpartum depression and anxiety received the most likes, with all 31 participants liking the video. Three posts garnered 30 likes; they were a video about understanding infant cues, a video with ideas for communication activities during wake windows, and a video about how to respond when the cries of your baby are too much. Eleven posts received 29 likes, including but not limited to a video about what a baby’s brain needs in the first few weeks, a video about strategies to calm a baby, a video about the myth of spoiling a baby through too much holding, a video about how to talk to a young infant, and a video about how to play with a young infant. Notably, videos that were posted toward the end of the 18-week intervention period received fewer (23) likes. These videos included 2 videos about babies and screen time and 2 videos about communicating with babies in a bilingual family. The post that received the least number of likes (20) was posted last and was a video titled “Things That Might Cause Concern in the First Year.” On average, viewership of each post averaged 85% (26.48 likes out of a possible 31 likes); mean=26.48 likes per video, range=20-31, mode=27. The number of comments per post ranged from 0 to 20 (mean 5.69, mode 3, 4). The post with the greatest number of comments (20) was the video titled *You Can’t Spoil a Baby*. A video about postpartum depression and anxiety had 16 comments, along with a video about understanding your baby’s cues. The post with the fewest (0) comments was a video about communication skills to expect during the first year. Videos with 1 comment each included a video about research on routines for babies, a video about baby signs, a still post with a baby sign guide, and a video about how to read to a baby.

We also analyzed the number of likes per participant and the number of comments per participant. Participants were asked to like a post as confirmation that they engaged with the content; 13% (4 out of 31) of participants watched all video posts, and 84% (26 out of 31) of participants watched more than half of the videos (mean likes per participant=49.55, range=21-58, mode=57). The number of comments per participant was also gathered to understand more about participant engagement; mean=10.65, range=0-48, and mode=2. One participant engaged with 48 comments, 5 participants commented more than 20 times, and 5 participants did not comment at all throughout the course of the intervention.

#### Caregiver Postsurvey

Following the intervention phase, the postsurvey was administered to gather caregiver perspectives of the intervention (see Multimedia Appendix 1). Results showed that the statements most frequently agreed upon were “The information shared was interesting” (27/31 [87%] strongly agreed), “The information and language used were clear and easy to understand” (27/31 [87%] strongly agreed), and “The videos helped me feel confident that I can help my child’s language learning” (27/31 [87%] strongly agreed). The statements with the lowest level of agreement were “Watching the videos changed my routines with my child” (16/31 [52%] strongly agreed; 9/31 [29%] agreed) and “I shared the videos with friends and family” (16/31 [52%] strongly agreed).

When asked, “What specific topics of videos did you find the most helpful or interesting?,” the majority of caregivers (26/31, 84%) selected *baby gestures*, followed by *early cues* and *brain development* (23/31, 74%). The topic with the lowest number of votes was *shared attention* (13/31, 42%).

### Qualitative Results

#### Participant Instagram Comments

##### Thematic Analysis

Through the qualitative data analysis process described in the Data Analysis section, initially 8 themes emerged: *venting*, *sharing*, *supporting each other*, *learning*, *practicing strategies*, *positive feedback*, *changes made*, and *questions*. At this stage, each of these themes was assigned a color, and each researcher separately coded the comments by highlighting them with the assigned color. After this initial coding process, the team met again to review each other’s coding for discrepancies and to define the codes with more specificity. The team agreed that some changes to the codes were necessary to more clearly describe the data. Specifically, *venting* was renamed as *sharing a challenge*. *Sharing* was changed to *sharing positive experiences* and was defined more clearly as sharing about their babies’ growth, milestones, and/or positive personal experiences but did not include discussing a challenge. *Learning*, *practicing strategies*, and *changes made* were merged under one code titled *learning through BabyTok*. The code titled *nonaligned/unclear intent* was added to label comments that did not match the topic of the video or post. Additionally, the code of *general comment* was added to describe a brief comment that expanded on the content topic. The resulting 8 themes are labeled and described in Table 4.

**Table 4:** Themes from analysis of participant Instagram comments.

Theme	Definition	Example comment
Sharing positive experiences	Participants shared updates on their babies’ growth, milestones, and/or positive personal experiences.	“I love talking to my baby and reading him my favorite books! He’s only three months and is babbling SO much.”
Sharing a challenge	Participants’ comments were related to struggles and/or venting about challenges related to the baby or about being a caregiver.	“The first couple of weeks after postpartum I was alone and had to deal with the baby and myself. On one particular night she was crying non stop. I tried everything I could to try and comfort her but nothing helped and I cried with her...it felt like such a sad moment to me because I felt hopeless and without sleep.”
Learning through BabyTok	Participants commented about learning something new, using a specific strategy, and/or making a specific change because of information learned from the videos.	“Through this page I’ve started to narrate what I do with my baby. If I’m making her a bottle, if I’m changing her, just making small talk, etc. and she understands and starts to coo which is very very cute! I like that she’s able to distinguish my voice from others and starts to move around.”
Supporting each other	Participants made comments that were supportive of other participants.	“I totally understand some days I feel like this as well. Keep pushing mama you got this.”
Positive feedback	Participants made comments that were compliments, positive statements, and/or emoji related to the videos, posts, topics, and/or BabyTok in general.	“I love this ❤.”
General comment	Participants made comments that were statements related to the content that expanded on the topic.	“Reading helps with cognitive growth, especially pictures.”
Nonaligned/unclear intent	Participants’ comments were unrelated, nonsensical, had unclear intent, and/or did not align with the content of the video or post.	“I can really relate to this.” (This comment was in response to a TikTok video repost with Ms Rachel modeling baby signs.)
Questions	Participant comments were questions for the BabyTok facilitator.	“During my babies wake window I try doing tummy time but she flat out refuses to exercise or stay with her arms crossed for more than 5 minutes. Any tips or suggestions?”

Instagram comments findings based on Table 4 themes are described in more detail below. Additionally, key participant comments are included to help contextualize these results.

##### Sharing Positive Experiences

Throughout the intervention, participants shared positive experiences with each other, such as sharing about their babies’ growth, milestones, and/or positive personal experiences related to caregiving of their baby. One participant shared the following about her baby when commenting on a video about understanding your baby’s cues:

I’ve been experiencing this lately! My daughter just turned two months & she’s really good at letting me know with her facial expressions when she’s hungry or wants to be held so she can nap.

Another participant commented the following on a video about free things to do in the community with the baby:

When we run errands I usually like to point stuff out & tell her what it is. Like if we’re at the grocery store I’ll say, this is an apple or we need beef. Just to have a little conversation with her & show the item.

##### Sharing a Challenge

Participants shared comments related to the challenges faced as a caregiver of an infant. When responding to one video post about postpartum depression and anxiety, there were several participants who shared their struggles. One participant commented, “Most times i feel like I’m loosing myself 
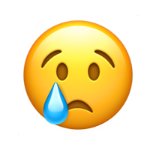
...postpartum stress is not really easy.” On a video posted about wake window routines a participant commented, “Right now im so tired i talk to her and watch her sisters play. I’m exhausted lol not much energy I have...”

##### Learning

Participants made comments related to learning from the BabyTok Instagram page. One participant commented about changes she has made because of the BabyTok intervention:

Through this page I’ve started to narrate what I do with my baby. If I’m making her a bottle, if I’m changing her, just making small talk, etc. and she understands and starts to coo which is very very cute!

Another participant commented that she plans to try a playtime strategy that was introduced in a video titled *Using Toys for Tracking*. Her comment was, “I’m going to start incorporating this during my babies wake time. thank you!”

##### Supporting Each Other

Participants also showed support for each other with comments like, “I totally understand some days I feel like this as well. Keep pushing mama you got this,” and “Happy new week fellow moms!” Participants sometimes shared strategies directly with another participant to be helpful. One participant was worried about her baby getting a flat head due to always sleeping on one side, and another participant commented the following:

Does she sleep facing a certain way? If so, you can try putting her head where her feet usually are. Then if she sleeps facing out the same way, she is now on her other side. (If that makes sense.) My niece also needed chiro and PT due to tight neck muscles on one side that led to her always wanting to face only one way.

##### Positive Feedback

Participants provided positive feedback on the content and/or the BabyTok Project Instagram page in general with compliments, supportive statements, and/or emojis. One such comment was made on a video about reading with a baby at different times throughout the day. The participant wrote, “This project has really helped me and my newborn.” Examples of other positive feedback comments that were made throughout the project were, “Thank you for sharing,” “Interesting stuff,” and “I love this ❤.”

##### General Comment

Participants also made general comments that expanded on the content. For example, when commenting on a video about the benefits of reading books, a participant wrote, “Reading helps them with cognitive growth, especially pictures,” and when commenting on a video about using wait time as a strategy, another participant stated, “Language is so important! Babies need that and also room to respond!”

##### Nonaligned or Unclear Intent

There were comments that were not aligned with the content or had unclear intent. One such comment was made on a video about free community routines. The participant’s comment was, “I can relate.” Another comment that was not aligned with the content was made on a video about the stress and anxiety that caregivers feel when babies cry for extended periods of time. The comment was, “Little miracles, there are such a joy.”

##### Questions for BabyTok

Participants also asked questions in the comments for the BabyTok Project’s facilitator, who was the first author. One such question was related to things to do with a baby during baby’s wake window. The participant asked:

During my babies wake window I try doing tummy time but she flat out refuses to exercise or stay with her arms crossed for more than 5minutes. Any tips or suggestions?

Another participant asked a question about a video that was posted about choosing books with real human faces to read to babies. The participant asked, “Could my baby enjoy this at two months?”

#### Caregiver Postsurvey

##### Thematic Analysis

Three open-ended questions were included in the caregiver postsurvey. They were “Please share any feedback or advice you have for us about the videos,” “What was your main takeaway from the content posted,” and “Please share what additional topics you might like information about in future videos.” The main themes of *feedback and/or advice*, *main takeaways*, and *future videos* were derived from the questions in the survey. Through the template analysis process [10], the second and third authors individually studied the participant answers. The codes that emerged were *positive feedback*, *negative feedback*, *advice*, *empower*, *communication*, *strategy*, *informative*, *affirmation*, *change*, *postpartum*, *feeding*, *different ages/milestones*, and *delivery of content*. The two authors continued to analyze the data and met several times until consensus was reached. During this process, each author took notes about the coding process, thinking about the following questions: Did the codes fit well? Did new themes emerge? Did some codes overlap? Did the codes describe the data accurately? The main code of *future videos* was integrated into the *feedback/suggestions* code because there was crossover between the themes that emerged for each of these codes. Two main codes remained, and the subcodes for each main code were formalized and organized to more accurately describe the data. The code *main takeaway* was given the subcodes of *new information*, *empowering/affirming*, *strategy used/change made*, and *null/negative effects*. The main code, *feedback/suggestions*, was given the subcodes of *general positive statement*, *delivery of content*, *different ages/milestones*, *pregnancy and/or postpartum*, and *unrelated topics*. The themes along with their subthemes are described in Table 5.

**Table 5:** Themes from analysis of postsurvey open-ended responses.

Theme			Definition		Example participant response
**Main takeaway**					
	New information	Caregivers reported learning new information from viewing the BabyTok Project videos.		“Learning two languages at the same time helps the brain to develop better, contrary to popular opinion that it may hinder language development.”	
	Empowering/affirming	Caregivers recognized their own impact on their baby and/or felt their parenting communications were making a difference.		“There is no such thing as a perfect parent. Also that you can take charge and help with the development of your baby.”	
	Strategy used/change made	Caregivers mentioned using a specific strategy and/or making a specific change because of information learned from the videos.		“I stopped letting my baby have screen time.”	
	Null effects	Caregiver responding with no comment or nothing learned from intervention.		“Nothing really.”	
**Feedback/suggestions**					
	General statement	Any general positive comment related to the intervention as a whole.		“I love this segment and truly hope it continues. As a new mom it’s just so helpful.”	
	Delivery of content	Any comment with advice related to how the intervention was delivered and/or the platform used for the intervention.		“...it would be nice if specific days were set aside for posting and not just randomly any day.”	
	Different ages/milestones	Caregiver suggestions that future videos include additional ages of development from during pregnancy to older childhood.		“I’d love to have the option for different chapters such as pregnancy through toddlerhood, elementary through middle school, etc.”	
	Unrelated topics	Caregivers mentioned other topics not related to early language development and communicating with the baby such as feeding the baby, teething, helping baby learn to walk, siblings, activities with baby, etc.		“I’d love topics such as starting solids, teething, and even fun activities to do.”	
	Null effects	Caregiver wrote N/A, no additional comment, not sure, or left the answer box blank.		“No additional comment.”	

Thematic analysis of the open-ended questions yielded key findings, which are described in detail below.

##### Main Takeaway

When answering the main takeaway question, participants gave answers showing that they learned new information. One participant answered by stating, “How my everyday interactions with my child is helping her learn and grow even if they feel small.” Another participant stated, “It is really important to play and talk with my baby.” Participants also described feeling empowered or affirmed. One participant wrote, “There is no such thing as a perfect parent. Also, that you can take charge and help with the development of your baby.” Additionally, participants described strategies learned from the intervention or a change made as a result of the intervention. For example, one participant wrote, “I’ve stopped letting my baby have screen time.” Another participant wrote, “I loved how you talked about building routines not schedules. The pressure that I lifted off of myself was huge. Routines are much easier to navigate with littles than schedules.” There was one null response from a participant who answered the main takeaway question with the response, “nothing really.”

##### Feedback/Suggestions

Participants were asked to share their feedback, advice, and ideas about future topics for the Babytok Project. Participants answered with general positive statements such as, “I love this segment and truly hope it continues. As a new mom it is just so helpful,” and “The project was really helpful for my infant and I.” Participants also answered with suggestions about content and delivery. One participant wrote, “Have them at a set time,” and another wrote, “...it would be nice if the specific days were set aside for posting and not just randomly any day.” Another participant answered with, “should do more videos.” One participant wrote that they would like to see more representation in videos stating, “Include more videos featuring Dads, Grandparents, and other caregivers to promote diversity and representation in parenting roles.” Participants also wrote that they would like videos in the future to include additional developmental ages and discuss developmental milestones. One participant wrote, “...going through all the stages of childhood would be a great direction for the page...creating a series or episodes for different stages would be great.” Likewise, another participant wrote, “I’d like it to continue to cover toddler age or at least the first year...” Participants also wrote that they were interested in receiving more information during pregnancy, with one participant writing, “I’d love to have access to something like this during pregnancy so I can also be prepared before baby is born...” Participants were also interested in more information on postpartum health, teething, baby foods, common health issues in infants, and information on motor skills. There were several (14) null answers with participants giving answers such as N/A, no additional comment, or leaving the response box blank.

## Discussion

### BabyTok Project Iterations

This investigation builds evidence for the BabyTok Project social media intervention to support caregiver knowledge of early communication development. Caregivers of infants reported high levels of satisfaction with the intervention, and data from the quantitative measure indicate significant pre-to-post growth in caregiver knowledge of early child development, although these findings should be viewed in the context of the quasi-experimental design that does not have a control group. Given the correlation between caregiver knowledge of child development/language supporting strategies and the quality of very early interactions [17,32], this is a positive indication that light-touch interventions like the BabyTok Project may increase caregiver knowledge. Caregiver knowledge is known to mediate caregiver-child interactions, although a randomized controlled trial is needed to explore potential impacts of the BabyTok Project on real-time exchanges between infants and caregivers.

This iteration of the BabyTok Project began before 3 months of age and was longer than the toddler version of the BabyTok Project investigation with parents of toddlers (22 weeks compared to 8 weeks). These factors might explain the more sizeable increases in SPEAK-R scores in addition to the added statistical power achieved through a larger sample size. Although we did see decreases in engagement toward the end of the 22-week period, slightly longer interventions might be needed to ensure that caregivers are receiving enough of the content to increase their knowledge about early development.

Compared to other app-based interventions in which caregivers interact with in-app features and researchers, the BabyTok Project included a social element between caregivers in small groups. As we plan to expand and scale the project, we will retain the group nature of the intervention to encourage interactions between caregivers in the comments section on the videos. Because we monitor the comments, we are able to correct any false or misleading information that may appear. The benefit of this approach is that it enables caregivers to encourage one another, share ideas, and ask questions. Although it is not possible to disentangle the contribution of the “social” element apart from the content of the videos themselves, we believe it may be an important component that keeps parents engaged and that may build caregiver confidence by sharing information with one another. This approach does, however, complicate the available engagement metrics because watch time data are not available from private pages. Researchers who use social media platforms will need to balance the nuance of information available with privacy and confidentiality requirements of research.

Social media–based video content reaches caregivers on platforms like Instagram, on which they already spend time and search for information. Because social media–based interventions in this area are rare, we do not have many other contexts for comparison as it relates to the video watch rates and amount of engagement. Compared to our own prior work, however, this study had the highest percentage of videos watched, perhaps due to stronger implementation infrastructure with a research team member helping to distribute and follow up with participants every 2 weeks. This could be an important feature to ensure that the videos reach the intended audience, rather than relying on the algorithm alone to alert the users to new content. In fact, participants in past versions have noted that the algorithm did not return them to the page’s content, so the direct messaging was essential. Contact with the research team also provided a checkpoint to make sure that participants were doing well, and in the future, it could also be an avenue to weave in a place for caregivers to ask questions about their child’s learning/development if concerns arise, or even to promote the use of very early developmental screeners.

### Implications for Other Caregiver-Focused Social Media Interventions

Participants in this study had several suggestions that are relevant to other social media–based studies outside of early language development. First, they recommended a predictable video release schedule so that they know when the videos are going to be posted (ie, Mondays, Wednesdays, and Fridays at 12 pm), although they did not specify preferred days and times for video distribution. Across studies, participants appreciated the direct messaging of content rather than relying on them to seek out the page or for the algorithm to deliver it. Participant feedback to include videos of multiple types of caregivers (ie, grandparents, aunts/uncles, and siblings) in the videos is also an important implication for other social media–based interventions for caregivers.

### Limitations and Future Directions

Although the findings from this pilot study are promising, there are several limitations to note. Our pilot sample was small, and we did not verify the identity of the participants with a synchronous virtual meeting. We followed the bot and masquerader procedures described above, but verifying identities in a virtual meeting over Zoom would have been ideal and will be used in future investigations. First, it would enable us to ensure that the participants are caregivers of infants and not masqueraders on the internet. Second, it would enable us to meet and connect with participants in a more personal way that will be essential in a longer-duration randomized controlled trial that includes follow-up data collection and other measurements (ie, LENA [Language Environment Analysis] samples, observations, etc).

It is also important to note that our pool of participants had higher levels of education than one might expect from a low-income sample. This could indicate that caregivers with higher levels of education might have self-selected into the study. In addition, the pre-post quasi-experimental design precludes causal conclusions about the ability of the intervention to create change in caregiver knowledge. It is possible that caregivers gained information from other sources during the time they were in the study or that there was a testing effect from taking the SPEAK-R twice. It is also important to view the usage/engagement metrics within the context of the approach used in this study. The videos watched do not reflect the “organic” rate that we may have achieved with little researcher support and outreach. While we do not view this as a limitation, per se, we do view it as an important implementation feature that is key for social media interventions that use short-form videos. In addition, the measure used in this study, while not developed by the research team, does not appear to have external validation studies. Still, it is one of the only tools that assesses caregiver knowledge of early communication.

### Conclusions

Multiple studies with different sets of caregivers endorse the BabyTok Project as a means to learn about children’s early communication. Given the established feasibility and acceptability of the approach and preliminary evidence of changes in knowledge, future studies will examine whether the approach can enhance caregiver-child interactions during infancy and, as a result, if children whose parents received the intervention have stronger language outcomes than a control group. If future efficacy testing demonstrates positive changes in caregiver-child outcomes, the BabyTok Project and interventions like it could fill a persistent need for preventative, universal interventions to promote children’s early language skills.

## Appendix

Multimedia Appendix 1 Postintervention feedback tables.

## Abbreviations

KIDI: Knowledge of Infant Development Inventory
LENA: Language Environment Analysis
SPEAK-R: Survey of Parent/Provider Expectations and Knowledge–Research
TANF: Temporary Assistance to Needy Families
WIC: Women, Infants, and Children

## References

[1] Huber E, Corrigan NM, Yarnykh VL, Ramírez NF, Kuhl PK (2023). Language experience during infancy predicts white matter myelination at age 2 years. J Neurosci.

[2] Fernald A, Marchman VA, Weisleder A (2013). SES differences in language processing skill and vocabulary are evident at 18 months. Dev Sci.

[3] Huttenlocher J, Waterfall H, Vasilyeva M, Vevea J, Hedges LV (2010). Sources of variability in children's language growth. Cogn Psychol.

[4] Romeo RR, Leonard JA, Robinson ST, West MR, Mackey AP, Rowe ML, Gabrieli JDE (2018). Beyond the 30-million-word gap: children's conversational exposure is associated with language-related brain function. Psychol Sci.

[5] Romeo RR, Leonard JA, Grotzinger HM, Robinson ST, Takada ME, Mackey AP, Scherer E, Rowe ML, West MR, Gabrieli JD (2021). Neuroplasticity associated with changes in conversational turn-taking following a family-based intervention. Dev Cogn Neurosci.

[6] Noble KG, Magnuson K, Gennetian LA, Duncan GJ, Yoshikawa H, Fox NA, Halpern-Meekin S (2021). Baby’s first years: design of a randomized controlled trial of poverty reduction in the United States. Pediatrics.

[7] Johnson SB, Riis JL, Noble KG (2016). State of the art review: poverty and the developing brain. Pediatrics.

[8] Greenwood CR, Schnitz AG, Carta JJ, Wallisch A, Irvin DW (2020). A systematic review of language intervention research with low-income families: a word gap prevention perspective. Early Child Res Q.

[9] Heidlage JK, Cunningham JE, Kaiser AP, Trivette CM, Barton EE, Frey JR, Roberts MY (2020). The effects of parent-implemented language interventions on child linguistic outcomes: a meta-analysis. Early Child Res Q.

[10] Suskind DL, Leung CYY, Webber RJ, Hundertmark AC, Leffel KR, Suskind E, Hernandez MW, Graf E (2017). Development of the survey of parent/provider expectations and knowledge (SPEAK). First Lang.

[11] Alper R, Luo R, Mogul M (2023). Duet: an exploratory language intervention for toddlers in low-income households. Infants Young Child.

[12] Landry SH (2020). Supporting parents with the Play & Learning Strategies Program (PALS). Int J Birth Parent Educ.

[13] Luo T, Li M, Williams D, Phillippi S, Yu Q, Kantrow S, Kao Y, Celestin M, Lin W, Tseng T (2021). Using social media for smoking cessation interventions: a systematic review. Perspect Public Health.

[14] Cao B, Gupta S, Wang J, Hightow-Weidman LB, Muessig KE, Tang W, Pan S, Pendse R, Tucker JD (2017). Social media interventions to promote HIV testing, linkage, adherence, and retention: systematic review and meta-analysis. J Med Internet Res.

[15] Orchard LJ, Nicholls W (2022). A systematic review exploring the impact of social media on breastfeeding practices. Curr Psychol.

[16] Singletary B, Jiang H, Wilberforce WG, Avelar D, Strother-Garcia K, Justice LM (2025). Examining early vocabulary growth trajectories in late talkers in a low-income longitudinal sample. Infancy.

[17] Leung CY, Suskind DL (2020). What parents know matters: parental knowledge at birth predicts caregiving behaviors at 9 months. J Pediatr.

[18] Baralt M, Mahoney AD, Brito N (2020). Háblame Bebé: a phone application intervention to support Hispanic children's early language environments and bilingualism. Child Lang Teach Ther.

[19] Baralt M, Mahoney AD, Brito N, Larson A (2022). Nurse Home Visitors’ use of the Háblame Bebé app to support mother-child interactions in their home language during COVID. Home Visiting Applied Research Collaborative.

[20] Larson AL, Baralt M, Hokenson J, Hammer CS, Barrett T, DeVilbiss N (2022). A randomized controlled trial assessing the effectiveness of the Háblame Bebé mobile application with Spanish-speaking mothers experiencing economic hardship. Am J Speech Lang Pathol.

[21] Larson AL, Caballero R, Snyder P, Griebel S, Valentin R, Bourret C (2023). Gathering perspectives on video- and app-based language instruction: a technology-based coaching intervention to increase Spanish-speaking Latino caregivers' use of naturalistic language intervention strategies. Perspect ASHA SIGs.

[22] Mertens E, Ye G, Beuckels E, Hudders L (2024). Parenting information on social media: systematic literature review. JMIR Pediatr Parent.

[23] Frey E, Bonfiglioli C, Brunner M, Frawley J (2022). Parents' use of social media as a health information source for their children: a scoping review. Acad Pediatr.

[24] Moon RY, Mathews A, Oden R, Carlin R (2019). Mothers' perceptions of the internet and social media as sources of parenting and health information: qualitative study. J Med Internet Res.

[25] Pretorius K, Johnson KE, Rew L (2019). An integrative review: understanding parental use of social media to influence infant and child health. Matern Child Health J.

[26] Romano M, Perez K, Abarca D (2024). The BabyTok project: examining the feasibility and acceptability of a light-touch social media project for infant-toddler teachers. Early Child Educ J.

[27] Romano M, Abarca D, Baehman F (2025). A low-cost, social media–supported intervention for caregivers to enhance toddlers’ language learning: mixed methods feasibility and acceptability study. JMIR Pediatr Parent.

[28] Lawlor J, Thomas C, Guhin AT, Kenyon K, Lerner MD, Drahota A (2021). Suspicious and fraudulent online survey participation: introducing the REAL framework. Methodol Innov.

[29] Suskind DL, Leung CYY, Hundertmark AC, Ladner PH, Leffel KR, Miller K (2019). Impacting low-SES caregiver knowledge, language behaviors, and infant vocabulary skills through a web-based pediatric primary care intervention: a randomized controlled trial. Pediatrics.

[30] Gaudreau C, Levine D, List JA, Suskind DL (2025). Introducing the SPEAK: a scalable, computer-adaptive tool to measure knowledge of early human development. National Bureau of Economic Research.

[31] Alshurafa N, Jain J, Alharbi R, Iakovlev G, Spring B, Pfammatter A (2018). Is more always better? Discovering incentivized mHealth intervention engagement related to health behavior trends. Proc ACM Interact Mob Wearable Ubiquitous Technol.

[32] Instagram.

[33] Cohen J (1988). Statistical Power Analysis for the Behavioral Sciences. 2nd ed.

[34] Brooks J, McCluskey S, Turley E, King N (2015). The utility of template analysis in qualitative psychology research. Qual Res Psychol.

[35] Nowell LS, Norris JM, White DE, Moules NJ (2017). Thematic analysis: striving to meet the trustworthiness criteria. Int J Qual Methods.

