# Supplementary material for: A Social Media Intervention to Increase Caregiver Knowledge of Early Infant Communication: Multiple Methods Intervention Development and Pilot Study
Source: JMIR Pediatr Parent. 2026 Sep 30;9:e93171. doi: 10.2196/93171 (PMC13669702; doi:10.2196/93171)
Supplement: Supplementary file 1 [file pediatrics_v9i1e93171_app1.docx]

**Table S1.** Postintervention feedback survey results (n=31).

| **Statement** | **Strongly disagree** | **Disagree** | **Neutral** | **Agree** | **Strongly agree** |
| --- | --- | --- | --- | --- | --- |
| I enjoyed watching the weekly posts. | 3% |  |  | 23% | 74% |
| Watching the videos changed my interactions with my child. |  |  | 3% | 32% | 65% |
| Watching the videos changed my routines with my child. |  |  | 19% | 29% | 52% |
| The information shared was interesting. |  |  |  | 13% | 87% |
| The information shared was useful to my everyday life. |  |  |  | 19% | 81% |
| The information and language used were clear and easy to understand. |  |  |  | 13% | 87% |
| Accessing and viewing the BabyTok videos was straightforward and user-friendly. |  |  | 3% | 13% | 84% |
| I learned something new from the videos. |  |  |  | 16% | 84% |
| The number of videos posted was just right—not too many and not too little. |  |  |  | 32% | 68% |
| The length of the videos was just right—not too long and not too short. |  |  |  | 26% | 74% |
| I would share this social media page with other caregivers with infants or those who are expecting. |  | 3% | 10% | 6% | 81% |
| I shared the videos with friends and family. | 3% | 10% | 19% | 16% | 52% |
| The videos helped me feel confident that I can help my child’s language learning. |  |  | 3% | 10% | 87% |

**Table S2.** What specific topics of videos did you find the most helpful or interesting?

| Brain development | 24 (75%) |
| --- | --- |
| Pep talk/affirmations | 18 (56%) |
| Early cues | 24 (75%) |
| Routines | 17 (53%) |
| Books | 19 (59%) |
| Specific strategies | 15 (47%) |
| Myth busting | 15 (47%) |
| Baby sounds and vocalizations | 23 (72%) |
| Shared attention | 13 (41%) |
| Baby gestures | 27 (84%) |
